# Identification and Developmental Profiling of microRNAs in Diamondback Moth, *Plutellaxylostella* (L.)

**DOI:** 10.1371/journal.pone.0078787

**Published:** 2013-11-13

**Authors:** Pei Liang, Bing Feng, Xuguo Zhou, Xiwu Gao

**Affiliations:** 1 Department of Entomology, China Agricultural University, Beijing, P. R. China; 2 Department of Entomology, University of Kentucky, Lexington, Kentucky, United States of America; University of British Columbia, Canada

## Abstract

MicroRNAs (miRNAs) are a group of small RNAs involved in various biological processes through negative regulation of mRNAs at the post-transcriptional level. Although miRNA profiles have been documented in over two dozen insect species, few are agricultural pests. In this study, both conserved and novel miRNAs in the diamondback moth, *Plutella xylostella* L., a devastating insect pest of cruciferous crops worldwide, were documented. High-throughput sequencing of a small RNA library constructed from a mixed life stages of *P. xylostella,* including eggs, 1st to 4th (last) instar larvae, pupae and adults, identified 384 miRNAs, of which 174 were *P. xylostella* specific. In addition, temporal expressions of 234 miRNAs at various developmental stages were investigated using a customized microarray analysis. Among the 91 differentially expressed miRNAs, qRT-PCR analysis was used to validate highly expressed miRNAs at each stage. The combined results not only systematically document miRNA profiles in an agriculturally important insect pest, but also provide molecular targets for future functional analysis and, ultimately, genetic-based pest control practice.

## Introduction

MicroRNAs (miRNAs) are small (18–24 nucleotides, nt) genome-encoded non-coding RNAs (ncRNAs) and play crucial roles during the post-transcriptional gene expression in eukaryotes. By guiding the RNA-induced silencing complex (RISC) to bind to target “seed match” sites within the 3′ untranslated region (UTR) of mRNAs, miRNAs can suppress the translation of its target mRNA and hence silence its expression [1], [2]. Evidence showed that some miRNAs can also suppress the expression of its target mRNA by binding to the 5′UTR [3] or open reading frame [4], [5] of the mRNAs. It is estimated that though only 1% of the genomic transcripts in mammalian cells encode miRNA [6], nearly 50% of the encoded genes are regulated by miRNAs [7]. There is mounting evidence suggests that almost all known physiological and pathophysiological processes are negatively regulated by miRNAs, such as insect development (including cell development, wing development, muscle development, neurogenesis and cell apoptosis), host-pathogen interactions and immunity [8], [9].

Since the first miRNA was discovered in *Caenorhabditis elegans* in 1993 [1], [2], miRNAs have been identified in insects, vertebrates, plants and virus. To date, a total of 25141 mature miRNAs have been documented in 193 species, of which 25 species belong to the 6 insect orders, including Diptera (15 species), Hymenoptera (4 species), Homoptera (1 species), Lepidoptera (3 species), Coleoptera (1 species) and Orthoptera (1 species).

The diammondback moth, *Plutella xylostella* (L.), is a devastating lepidopteran pest of cruciferous crops worldwide, and the damage and management costs are estimated at $4–5 billion annually [10]. Extensive studies on the ecology and management of *P. xylostella* have been reviewed by Furlong, et al [11]. Recent transcriptome analyses and genome sequencing provide a unique opportunity to gain a molecular understanding of its adaptations to stressed environments [12]–[14]. Although Etebari, et al [15] identified a subset of miRNAs from the second instar larvae under parasitic stress; a comprehensive inventory of miRNAs in *P. xylostella* is lacking. In this study, conserved and novel miRNAs from all developmental stages in *P. xylostella* were inventoried systematically.

## Materials and Methods

### Ethics statement

*Plutella xylostella* strains used in this study were initially collected in Beijing in 2000, and have been maintained in our laboratory at the China Agricultural University for over 120 generations. No specific permit was required for the described field collections, and the location is not privately-owned or protected in any way. The species in the genus of *Plutella* are common agricultural pests and are not included in the “List of Endangered and Protected Animals in China”.

### RNA isolation and sequencing

*Plutella xylostella* larvae and adults were reared at 27±1°C, 70±10% RH, and a 16∶8 L: D photoperiod, as described previously [16]. Total RNA was isolated from the whole body homogenates of a sample mix, contained 50 mg of eggs, 1^st^ to 4^th^ instar larvae, pupae and adults, respectively, using TRIzol reagent (Invitrogen, Carlsbad CA, USA) following the manufacturer's instructions. Thirty five micrograms of total RNA were size-fractionated on a 15% TBE-Urea polyacrylamide gel. Small RNA populations of 18–28 nt were extracted, purified, and ligated to a 3′ linker and a 5′ linker using T4 RNA ligase (Ambion), and ligation products were used for SuperScript II reverse transcription (Invitrogen). PCR reactions were carried out using the RT primer and 5′ PCR primer. Linker and primer sequences are provided in additional file, Table S1. Amplified cDNA products were gel-purified and sent to BerryGenomics (Beijing) for high-throughput sequencing on an Illumina Hiseq2000.

### Bioinformatics analysis

A proprietary software package, ACGT101-miR v3.5 (LC Sciences, Huston, USA), was used for analyzing sequencing data generated. Reads with no matches to the proximal 11 nt of the 5′-adaptor were removed. Then the Reads mapped to the RepBase (v17, http://www.girinst.org) and Rfam (http://www.sanger.ac.uk/Software/Rfam/ftp.shtml) were removed before further analysis.

For the remaining unique sequences, various “mappings” were performed against pre-miRNA and mature miRNA sequences listed in the miRBase (v18, http://www.miRBase.org/) or *B. mori* genome (http://silkworm.genomics.org.cn/). First, unique sequences which mapped to insect pre-miRNAs in miRBase and these pre-miRNAs mapped to *B. mori* genome were identified as conserved mature miRNA. Second, for the unique sequences mapped to insect pre-miRNAs but the pre-miRNAs did not map to *B. mori* genome, if the unique sequences mapped to the *B. mori* genome and their extended sequences potentially formed hairpins, or if the unique sequences did not map to *B. mori* genome but mapped to known insect miRNAs, they were considered as potential miRNAs of *P. xylostella*. The unique sequences un-mapped to insect pre-miRNAs but mapped to *B. mori* genome and their extended sequences potentially formed hairpins were also considered as potential miRNAs. The second structures of selected pre-miRNAs were predicted by using RNAfold [17].

### μParafloTM miRNA microarray

To validate the predicted miRNAs, microarray was performed using a service provider (LC Sciences). The assay started from 2 μg total RNA samples which were 3′-extended with a poly (A) tail using poly (A) polymerase. An oligonucleotide tag was then ligated to the poly (A) tail for later fluorescent dye staining; two different tags were used for the two RNA samples in jual-sample experiments. Hybridization was performed overnight on a μParaflo microfluidic chip using a micro-circulation pump (Atactic Technologies) [18], [19]. On the microfluidic chip, each detection probe consisted of a chemically modified nucleotide coding segment complementary to conserved or predicted novel *P. xylostella* miRNA and a spacer segment of polyethylene glycol to extend the coding segment away from the substrate. The detection probes were made by *in situ* synthesis using synthesis using PGR (photogenerated reagent) chemistry. The hybridization melting temperatures were balanced by chemical modifications of the detection probes. Hybridization used 100 μL 6×SSPE buffer (0.90M NaCl, 60 mM Na_2_HPO_4_, 6 mM EDTA, pH 6.8) containing 25% formamide at 34°C. After RNA hybridization, tag-conjugation Cy3 and Cy5 dyes were circulated through the microfluidic chip for dye staining. Fluorescence images were collected using a laser scanner (GenePix 4000B, Molecular Device) and digitized using Array-Pro image analysis software (Media Cybernetics). Data were analyzed by first subtracting the background and then normalizing the signals using a LOWESS filter (Locally-weighted Regression) [20]. For two color experiments, the ratio of the two sets of detected signals (log2 transformed, balanced) and p-values of the t-test were calculated; differentially detected signals were those with less than 0.01 p-values. Hierarchical clustering was carried out using the TIGR MeV (MultiExperiment Viewer) v4.1 software, http://www.tm4.org/mev.html [21].

### Quantitative RT-PCR

Twelve differently expressed miRNAs at various developmental stages were selected according to the initial microarray results and further verified using quantitative RT-PCR (qRT-PCR). Total RNA was extracted from 50 mg of eggs, 1^st^ to 4^th^ instar larvae, pupae and adults, respectively using TRIzol reagent as described previously. First strand cDNA was synthesized from 2 μg of total RNA using miScript II RT kit (Qiagen) following manufacturer's instructions. The qRT-PCR reaction consisted of 1 μL of diluted cDNA, 10 μL of SYBR Green Master Mix (miScript SYBR Green PCR Kit, Qiagen, USA) and1 μL of 10 μM of forward and reverse primer in 20 μL total volume. The forward primers were listed in table 1, and the universal reverse primer was supplied in miScript SYBR Green PCR Kit (Qiagen). The PCR reaction was conducted on an Applied Biosystems 7500HT Real-Time PCR System under the following conditions: 15 min template denaturation at 95°C, followed by 40 cycles of 94°C for 15 s, 55°C for 30 s, and 60°C for 34 s followed by the melting curve (68°C−95°C). Melting curves for each sample were analyzed after each run to check the specificity of amplifications. Three biological replicates with three technical replications were conducted for each qRT-PCR. A combination of three selected most stable miRNAs PN-isc-miR-276_R+1, PN-api-miR-9a_L+1 and PN-bmo-miR-279d_L-117TC (unpublished data) were used as endogenous references for normalization (Table 1).

**Table 1:** Primers used for qRT-PCR analysis.

	Gene name	Forward Primer Sequence (5′-3′)
Selected miRNAs	bmo-miR-989_R+1	GTGTGATGTGACGTAGTGGAAG
	bmo-miR-210_L+1R+2	CTTGTGCGTGTGACAGCGGCTAT
	bmo-miR-307_R+3	TCACAACCTCCTTGAGTGAGCGA
	bmo-let-7_R−1	TGAGGTAGTAGGTTGTATAG
	bmo-miR-100	AACCCGTAGATCCGAACTTGTG
	bmo-miR-750_R+210TA	CCAGATCTAACTTTCCAGCTCA
	PC-5p-3130	ATCCTGGCAGGGTCGCCA
	PN-cqu-miR-279_21AT	TGACTAGATCCACACTCATTTA
	bmo-miR-92b	AATTGCACCAATCCCGGCCTGC
	PN-api-miR-2c-3p	TCACAGCCAGCTTTGATGAGCAA
Reference miRNAs	PN-isc-miR-276_R+1	GCTGTCCGTTAGGAACTTCATAC
	PN-api-miR-9a_L+1	CCAGGATCTTTGGTTATCTAGC
	PN-bmo-miR-279d_L-117TC	GACGGGACTAGATTTTCACTCA

## Results

### Predicted *P. xylostella* miRNAs using *B. mori* genome as a reference

Solexa sequencing technology was used to identify miRNAs in the *P. xylostella*. A pooled small RNA library was constructed from the entire developmental stages of the insect (from eggs to adults). Sequencing yielded 4,620,660 reads, of which the reads without 3′ adaptor, or reads less than three copies or the length less than 15 nt after adaptor removal were all discarded (32.9%), and then the reads mapped to Rfam (8.9%) and Repbase (9.4%), as well as those mapped to *B. mori* mRNA (17.4%) were also filtered, leaving 1,450,828 (31.4%) reads were used for miRNA identification (Table 2). After various mapping, a total of 234 conserved and potential candidate miRNAs were identified (Table S2), of which 105 miRNAs were known ones from *B. mori* and other insects. The rest newly isolated ones considered novel miRNAs of *P. xylostella* were divided into two groups, prefixed with “PN-” and “PC-”, where “PN-” denotes sequences that mapped to pre-miRNA sequences from miRBase and “PC-” denotes sequences that mapped to *B. mori* genomic sequences with hairpin formation.

**Table 2:** Summary of *P. xylostella* small RNA data analysis.

Group of reads	Number of sequences	Mappable seq.(%)
Raw	4,620,660	100
Mapped to mRNA	802,454	17.4
Mapped to other RNAs (Rfam: rRNA, tRNA, snRNA, snoRNA and other)	410,350	8.9
Mapped to Repbase	433,743	9.4
Total mappable for miRNA	1,450,828	31.4
Mapped to miRBase (including nohit 1)	330,080	7.1
Mapped to *Bombyx mori* pre and mature miRNAs in miRBase and mapped to *B. mori* genome	300,679	6.5
Mapped to other insects pre and mature miRNAs in miRBase but not mapped to *B. mori* genome	102	0.0
Mapped to *B. mori* and other insects pre and mature miRNAs in miRBase but with new *B. mori* genome locations	10	0.0
Mapped to known other insects pre miRNAs and *B. mori* genome, within hairpins	2,585	0.1
Nohit 1	3,488	0.1
Unmapped to miRBase	1,143,974	24.8
Mapped to known other insects mature miRNAs but unmapped to *B. mori* genome	23,226	0.5
Unmapped to known other insects mature miRNAs but mapped to *B. mori* genome and within hairpins	4,523	0.1
Nohit 2	1,116,225	24.2
Mapped total	331,115	7.2
Nohit (including nohit 1 and nohit 2	1,119,713	24.2

**Figure 1:**
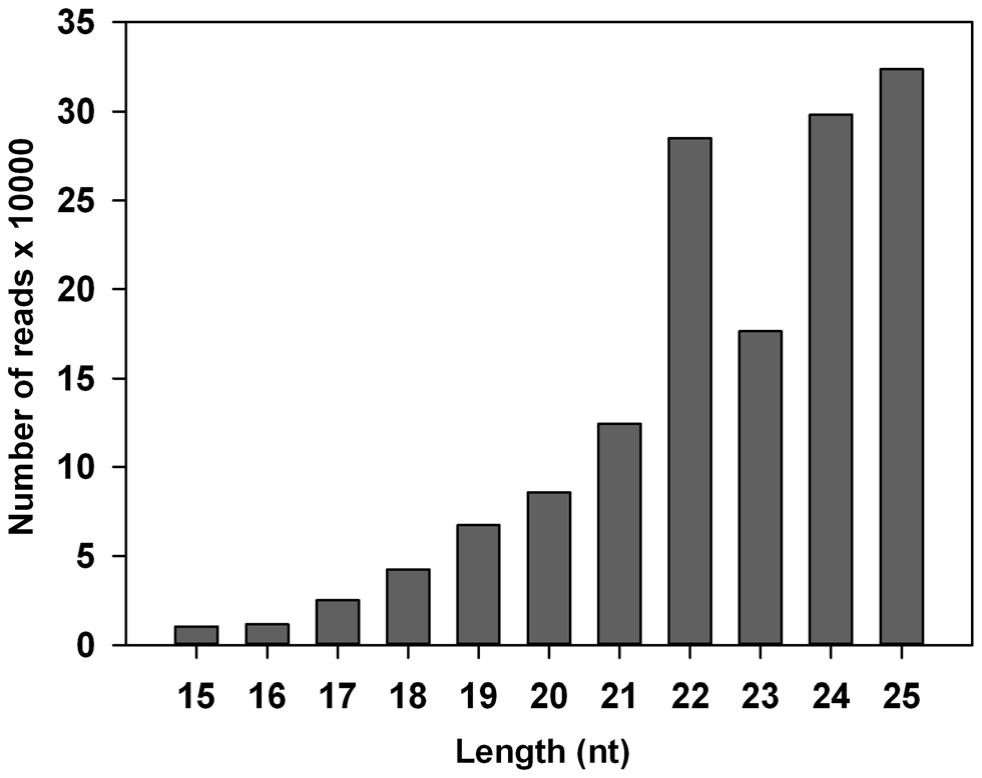
Length distribution of mappable reads obtained from *P. xylostella* deep sequencing. Reads with length >25nt were excluded from miRNA mapping.

As shown in Fig. 1, the length distribution of total mappable reads showed a peak at 22 nt which is the typical length of a mature miRNA, and the second peak appeared at 24–25 nt (Fig. 1). The length and copy number distribution of predicted miRNAs showed that more than 30% of miRNAs is 22 nt in length with the highest copy number (64.1%) among other miRNAs, and the miRNAs with 22–23 in length possessed more than 90% of reads. While the number of miRNAs with length 24–25 nt is less than 14.2% with even less copy number (<3.3%) (Table 3). According to the copy number, the five most abundant miRNAs were bmo-miR-1a, bmo-miR-8, bmo-miR-308, bmo-miR-100 and PN-bmo-miR-276*. A total of thirty three of most highly expressed miRNAs with copy number >1000 were listed in Table 4.

**Table 3:** Length distribution and copy number of the *P. xylostella* miRNAs.

miRNA Length	Number of miRNAs	%	Copy number	%
16	1	0.43	5	0.00
17	3	1.28	20	0.01
18	3	1.28	126	0.06
19	6	2.56	24	0.01
20	30	12.82	6575	2.95
21	24	10.26	6406	2.88
22	72	30.77	142775	64.12
23	62	26.50	59445	26.69
24	20	8.55	6332	2.84
25	13	5.56	1015	0.46
Total	234	100.00	222723	100.00

**Table 4:** The most abundant miRNAs in *P. xylostella* small RNA libraries.

miRNA	Sequence	Length	Copy Number	CG (%)	MFEI*
bmo-miR-1a	TGGAATGTAAAGAAGTATGGAG	22	36568	48.2	1
bmo-miR-8	TAATACTGTCAGGTAAAGATGTC	23	24384	50.0	0.7
bmo-miR-308	AATCACAGGATAATACTGCGAG	22	20786	44.7	0.9
bmo-miR-100	AACCCGTAGATCCGAACTTGTG	22	14286	45.8	0.5
PN-bmo-miR-276*	TAGGAACTTCATACCGTGCTCT	22	13433	43.7	0.7
bmo-miR-9a	TCTTTGGTTATCTAGCTGTATGA	23	6904	37.8	1.1
bmo-miR-277	TAAATGCACTATCTGGTACGACA	23	6725	50.8	1
bmo-let-7_R−1	TGAGGTAGTAGGTTGTATAG	20	6097	48.1	1
bmo-miR-279b	TGACTAGATCTACACTCATTGA	22	6049	41.6	1
bmo-miR-278	TCGGTGGGATCTTCGTCCGTTT	22	5612	56.3	0.8
bmo-miR-79_L-3R−1	ATAAAGCTAGATTACCAAAGCA	22	5093	59.0	0.8
bmo-miR-279a_R+3	TGACTAGATCCACACTCATCCA	22	5092	41.2	1
PN-tca-miR-3477-5p_R−1	TAATCTCATTTGGTAACTGTGAG	23	4796	39.6	0.9
bmo-miR-279c_R+1	TGACTAGATCCATACTCGTCTGC	23	4502	40.0	1
bmo-miR-750_R+210TA	CCAGATCTAACTTTCCAGCTCA	22	4077	50.6	0.8
bmo-miR-184_L−2	TGGACGGAGAACTGATAAGGGC	22	3540	52.4	0.8
bmo-miR-8*	CATCTTACCGGGCAGCATTAGA	22	3239	50.0	0.7
bmo-miR-14_R+1	TCAGTCTTTTTCTCTCTCCTAT	22	3193	40.0	1.2
bmo-miR-282_L-3R-2	TAGCCTCTCCTTGGCTTTGTCT	22	2655	48.2	0.9
bmo-miR-263a_R+3	AATGGCACTGGAAGAATTCACGGG	24	2610	48.4	0.8
bmo-miR-307_R+3	TCACAACCTCCTTGAGTGAGCGA	23	2292	56.3	0.8
PC-3p-68	TATTCGAGACCTCTGCTGATCC	22	2224	58.8	0.8
bmo-miR-12_R−1	TGAGTATTACTTCAGGTACTGG	22	1946	30.0	0.4
bmo-miR-305_R+1	ATTGTACTTCATCAGGTGCTCTGG	24	1863	56.0	1
bmo-miR-9c*_R+3	TCTTTGGTATCCTAGCTGTAG	21	1776	61.1	0.8
bmo-miR-13b	TATCACAGCCATTTTTGACGAGT	23	1761	50.0	1.1
bmo-miR-252	CTAAGTACTAGTGCCGCAGGAG	22	1545	43.5	0.9
bmo-miR-iab-4-5p	ACGTATACTGAATGTATCCTGA	22	1529	46.6	0.9
bmo-miR-2765	TGGTAACTCCACCACCGTTGGC	22	1482	54.7	0.9
bmo-miR-10_L+1	TACCCTGTAGATCCGAATTTGT	22	1454	47.6	1
bmo-miR-124_R-2	TAAGGCACGCGGTGAATGCCA	21	1292	51.8	0.8
PN-dme-miR-31a-5p_R-21TA	AGGCAAGATGTCGGCATAGCT	21	1209	53.3	1
bmo-miR-274_L-1R-2	TTTGTGACCGTCACTAACGGGCA	23	1144	49.5	0.8

* MFEI, minimum free energy index  =  -dG×100/length of the pre-miRNA sequence/GC%.

### Temporal expression of *P. xylostella* miRNAs at various developmental stages

**Figure 2:**
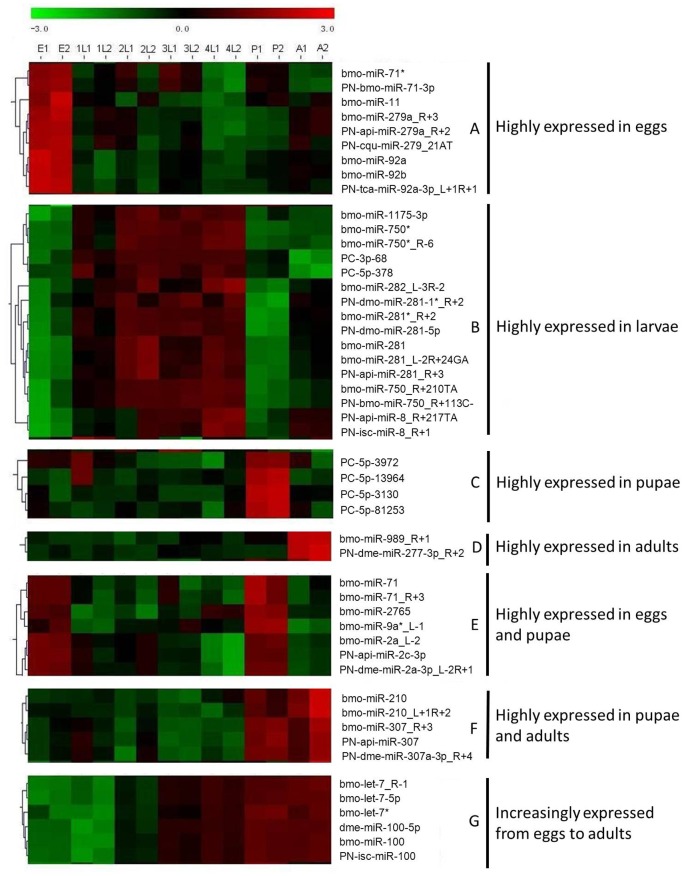
Microarray analysis of expression profiles of selected *P. xylostella* miRNAs at different developmental stages. After microarray hybridization and statistical analyses, a set of *P. xylostella* miRNAs differentially expressed in eggs, larvae, pupae and adults was identified. Two replicates and profiles clustering are presented for miRNAs significantly expressed among developmental stages. Microarray data were analyzed using the TIGR MeV (MultiExperiment Viewer) v4.1 software, http://www.tm4.org/mev.html, and one-way ANOVA. Color coding: red, up-regulated; black, mean; green, down-regulated.

The expression profiles of all 234 predicted miRNAs at various developmental stages were investigated using a customized microarray analysis. Among all the tested miRNAs, 143 miRNAs expressed evenly in eggs, larvae, pupae and adults, while the remaining 91 miRNAs were found differentially expressed at different developmental stages (Fig. S1). Of which nine miRNAs belong to four families, including miR-71, miR-11, miR-279 and miR-92, were highly expressed in eggs (Fig. 2A), and sixteen miRNAs from miR-1175, miR-750*, miR-281, miR-8 and four other families showed a significantly higher expression level in larvae stage but relatively lower expression level in eggs and pupae (Fig. 2B). There were four novel miRNA candidates (PC-5p-3972, PC-5p-13964, PC-5p-3130 and PC-5p-81253) and two miRNAs (bmo-miR-989_R+1 and PN-dme-miR-277-3p_R+2) were highly expressed in pupae and adults, respectively (Fig. 2C, 2D). A total of seven miRNAs were found highly expressed in both the eggs and the pupae, of which three miRNAs from miR-71 family and three from miR-2 family (Fig. 2E). In both pupae and adults, five miRNAs from both miR-210 and miR-307 families were found showed high expression level (Fig. 2F). There were six miRNAs increasingly expressed from eggs to adults, of which three belong to let-7 family and three miR-100 family (Fig. 2G).

**Figure 3:**
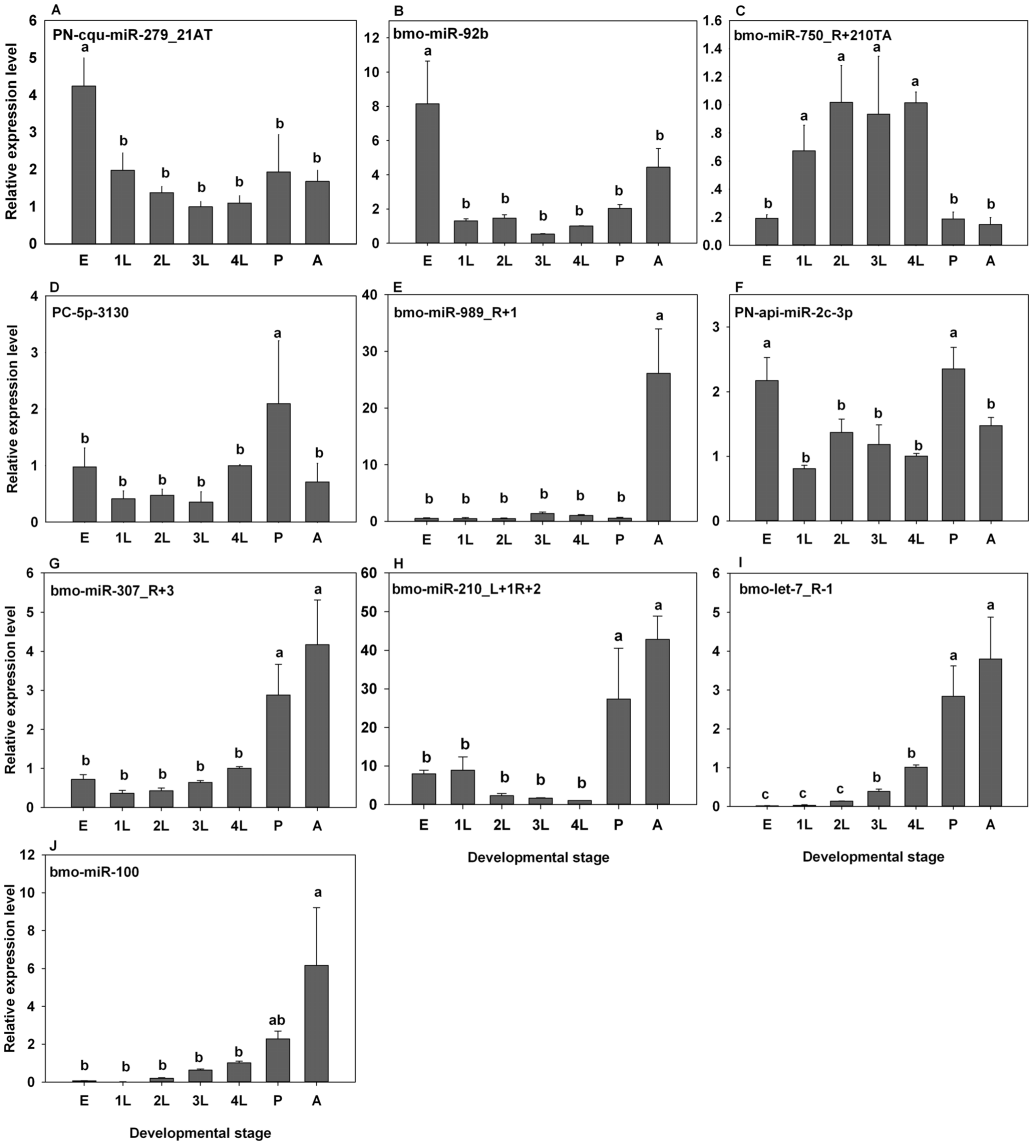
qRT-PCR analysis of differentially expressed *P. xylostella* miRNAs at various developmental stages. The expression of each miRNA was normalized to a panel of the three most stable miRNAs (Table 1, unpublished data). The relative miRNA expression at each developmental stage was normalized to the fourth instar larvae. Lowercase letters (a and b) represent significant differences (P<0.05).

To verify the microarray results described above, the relative expression levels of 12 differentially expressed miRNAs were further measured by qRT-PCR. Ten miRNAs showed similar expression patterns as those revealed by our microarray analysis. For example, the microarray showed that PN-cqu-miR-279_21AT and bmo-miR-92b were highly expressed in eggs, and the results of qRT-PCR showed that these two miRNAs were exclusively high expressed in eggs with 5.1- and 8.2-fold higher than that of in the fourth instar larvae, respectively (Fig. 3A, 3B). Similarly, the expression of bmo-miR-750_R+210TA in first to fourth instar larvae were 6.9- to 11.2-fold higher than that in eggs, and the expression of this miRNA in eggs showed no difference with that in pupae or adults (Fig. 3C). And the PC-5p-3130 and bmo-miR-989_R+1exclusively high expressed in pupae and adults respectively, and the expression level were 3.93- and 26.1-fold higher than that of in fourth instar larvae, respectively (Fig. 3D, 3E). While the PN-api-miR-2c-3p highly expressed in both eggs and pupae rather than in other stages (Fig. 3F), and both bmo-miR-210_L+1R+2 and bmo-miR-307_R+3 showed higher expression level in pupae (39.5- and 3.9-fold higher than that in 4^th^ instar larvae) and adults (42.9- and 4.2-fold higher than that in 4^th^ instar larvae) (Fig. 3G, 3H). Both the bmo-let-7_R−1 and bmo-miR-100 showed a increased expression from eggs to adults with significant difference in pupae and adults (Fig. 3I, 3J). The expression levels of miRNAs PN-bmo-miR-10*_R+1 and PN-aae-miR-87 detected by qRT-PCR inconsistent with that of the microarray results due to unknown reasons.

### Predicted *P. xylostella* miRNAs using its own genome as a reference

**Figure 4:**
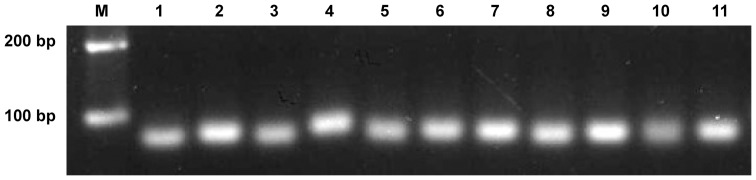
Comparative analysis of miRNA sequence similarity between *P. xylostella* and other insect species. As expected, *P. xylostella* miRNAs have the highest sequence similarity with Lepidoptera insects.

Most recently, the genome sequences of *P. xylostella* was released [14], and subsequently the *P. xylostella* miRNAs were predicted again based on its own genome sequences (http://iae.fafu.edu.cn/DBM/download.php). After mapping to the *P. xylostella* genome and miRBase (2012 August Release 19, http://www.mirbase.org/) using a software package ACGT101-miR v4.2 (LC Sciences, Houston, USA), a total of 348 miRNAs were identified (Table 5, Table S2). The majority of these miRNAs were conserved across insects, in which 222 miRNAs were found in other insects, including 189 in *B. mori*. The remaining 126 miRNAs were considered *P. xylostella* specific and prefixed with “PC” in their names, indicating they could map to the genome of *P. xylostella* within hairpin but they are not homologous to any known insect miRNAs. To validate these predications, eleven miRNAs including 5 novel and 6 conserved, were subjected to qRT-PCR analysis (Table S3). All of the predicted miRNAs were amplified using a cDNA template extracted from the fourth instar larvae (Fig. 4).

**Table 5:** Known and predicted *P. xylostella* miRNAs referencing to its own genome.

miRNA	Group	No. Unique miRNAs
Known miRNAs		
of *P. xylostella* in miRBase	Group 1a	0
of other insects, but novel to *P. xylostella*	Group 1b	31
Predicted miRNAs		
Mapped to known pre-miRNAs of other insects and *P. xylostella* genome; within hairpins	Group 2a	32
Mapped to known pre-miRNAs of other insects and *P. xylostella* genome; no hairpins	Group 2b	85
Mapped to known pre-miRNAs and miRNAs of other insects but unmapped to *P. xylostella* genome	Group 3a	70
Mapped to known pre-miRNAs of other insects but unmapped to *P. xylostella* genome	Group 3b	4
Unmapped to known miRNAs but mapped to *P. xylostella* genome and within hairpins	Group 4a	126
Total (Unique miRNAs)		348

**Figure 5:**
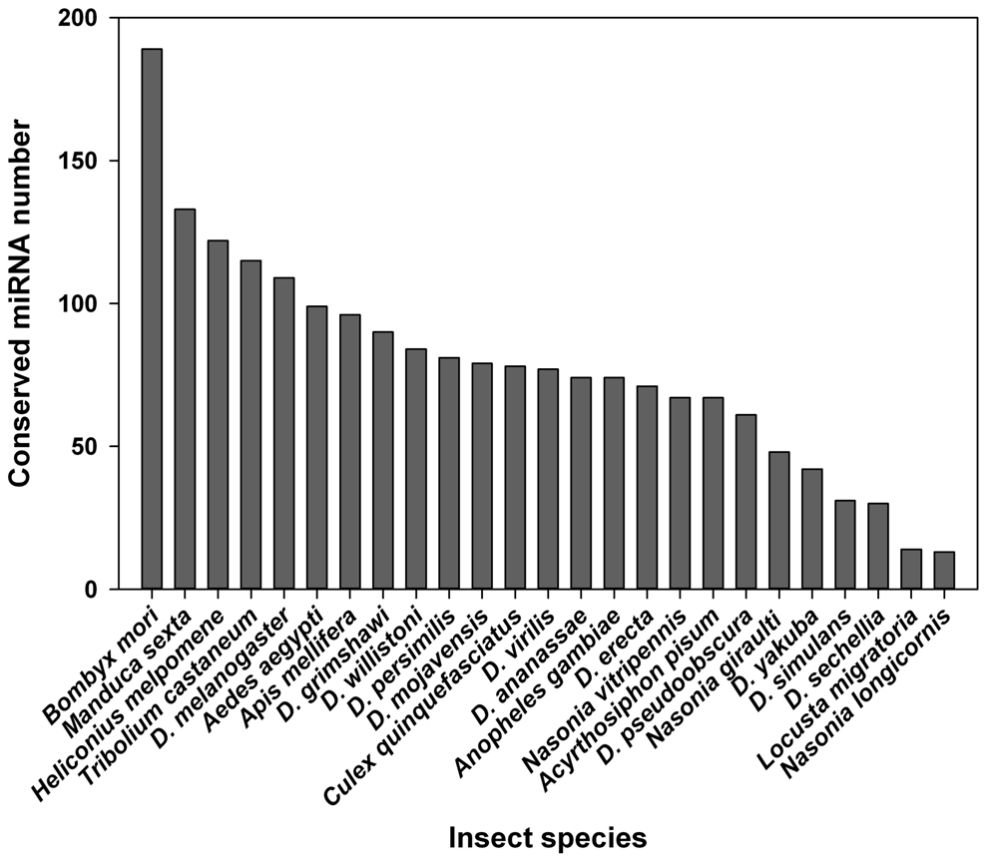
qRT-PCR validation of miRNAs predicted by the *P. xylostella* genome. cDNAs from the fourth instar larvae were used as the template. M: DNA marker. Lane 1: PC-5p-52_2942. Lane 2: bmo-miR-2755-3p. Lane 3: dme-miR-2a-3p_3ss18AT22GC23CT. Lane 4: dpu-bantam_R−1. Lane 5: PC-3p-174_795. Lane 6: bmo-miR-306a-5p_1ss1TC. Lane 7: bmo-miR-281-3p_L-2R+2. Lane 8: PC-3p-63_2387. Lane 9: PC-3p-61_2483. Lane 10: bmo-mir-6497-p5_1ss10CG. Lane 11: PC-5p-82_1775.

Up to date, miRNAs have been indentified and studied in 25 insect species. *Plutella xylostella* miRNAs are highly homologous to those of *B. mori* and two other lepidoprerans, *Manduca sexta* and *Heliconius melpomene* (Fig. 5). The most conserved region between *P. xylostella* pre-miRNA and other insects is the mature sequence and follows by the complementary sequence, whereas sequence far away from the mature region is highly varied. For example, mir-1a and mir-307 of *P. xylostella* showed very high identity to that of other insects, respectively, both at the mature and complementary regions (Fig S2, S3).

## Discussion

It has been demonstrated that miRNAs can affect almost all biological processes in insects [8]. The initial discovery of miRNAs in insects would lay the foundation for future functional characterization of these negative regulators. With the advent of the whole genome sequencing, miRNAs have been inventoried in 25 insect species including 12 *Drosophila* species [22], three mosquitos (*Anopheles gambiae*, *Aedes albopictus* and *Culex quinquefasciatus*) [23], [24], four hymenopterans (*Ahis mellifera*, *Nasonia virtipennis*, *N. giraulti*, and *N. longicornis*) [25], [26], three lepidopterans (*B. mori*, *M. sexta* and *Heliconius melpomene*) [27]–[29], one coleopteran (*T. castaneum*) [30], [31] and one hemipteran (*Acyrthosiphon pisum*) [32]. miRNAs in *Locusta migratoria* [33] and *Blattella germanica* [34] have also been documented. Most recently, miRNAs associated with parasitization by *Diadegma semiclausum* was reported in *P. xylostella* larvae [15]. These miRNAs were identified from the second instar larvae, and *B. mori* was used as a reference due to the lack of *P. xylostella* genome sequences.

In this study, a pooled small RNA library prepared from different development stages of *P. xylostella* (from eggs to adults) was sequenced. When using *B. mori* genome sequence as reference, a total of 234 miRNAs was identified, and when using the *P. xylostella*'s own genome data as reference, 348 miRNAs were identified, among which 120 miRNAs are overlapped. As a result, a total of 462 miRNAs were identified from various developmental stages in *P. xylostella*, of which 69 has been reported previously by Etebari, et al [15]. 174 miRNAs from the remaining 383 novel miRNAs were *P. xylostella* specific. These combined results are the first step toward understanding the roles of miRNAs in *P. xylostella* metamorphosis, physiological and behavioral adaptations to the environment.

To provide a fine-tuning of target gene expression, information regarding the temporal and spatial expression profiles of miRNAs is essential [35]. In *B. mori*, the dynamics of miRNA expression profiles in different tissues and developmental stages have been resolved, respectively [26], [36], [38]. Here, the temporal expressions of *P. xylostella* miRNAs were investigated using a microarray analysis. Among the 234 miRNAs tested, 143 were expressed evenly in eggs, larvae, pupae and adults, while the remaining 91 were differentially expressed. For example, a total of nine miRNAs from four families (miR-71, miR-11, miR-279 and miR-92) were highly expressed in eggs, suggesting their specific functions in regulating the embryogenesis and metamorphosis of *P. xylostella*. It has been reported that miR-11 regulate the apoptosis during embryogenesis [38], [39], while miR-279 determines olfactory neuron fate [40] in *Drosophila*. Similarly, miR-92 and miR-279 were found specifically expressed in the later embryos in *B. mori* [37]. During larval stage (from 1^st^ to 4^th^ instar), 16 miRNAs were significantly up-regulated, whereas, they were down-regulated in eggs and pupae, indicating that these miRNAs may be involved in the larvae-pupae transition. In *B. mori*, miR-8 and miR-281 were also highly expressed at larval stage [37]. As a highly conserved miRNA, miR-8 is associated with the neurodegeneration, wingless signaling, growth control and neuromuscular junction development [41]. High expressions of four novel miRNAs (PC-5p-3972, PC-5p-13964, PC-5p-3130 and PC-5p-81253) in pupae suggested that they may play important roles in metamorphosis from pupae to adult, while bmo-miR-989_R+1 and PN-dme-miR-277-3p_R+2 may have specific functions in adults because of their significantly elevated expression levels in adults. Similar to *B. mori*, both let-7 and miR-100 were up-regulated and gradually accumulated from eggs to adults in *P. xylostella*,

In total, there are 16, 16, 16, and 7 *P. xylostella* miRNAs specifically expressed in eggs, larvae, pupae, and adults, respectively. Zhang, et al [36] found that 106 of 354 validated miRNAs were expressed in all stages of *B. mori*, while the remaining miRNAs were egg- and pupa-specific, suggesting that insect miRNAs play a significant role in embryogenesis and metamorphosis.

Both microarray and qRT-PCR analyses have been used extensively to quantify miRNA expression. Results, however, are not always consistent. In this study, qRT-PCR analysis validated 10 out of 12 differentially expressed *P. xylostella* miRNAs identified by microarray analysis. Similarly, qRT-PCR analysis confirmed 6 out of 8 microarray-determined differentially expressed miRNAs in *B. mori* [36], suggesting that multiple tools, especially qRT-PCR analysis, should be used to accurately assess the expression level of miRNA.

## Supporting Information

Figure S1 **Microarray analysis of expression profiles of predicted** ***P. xylostella*** **miRNAs at different developmental stages.** After microarray hybridization and statistical analyses, all detectable *P. xylostella* miRNAs differentially expressed in eggs, larvae, pupae and adults were subjected to one-way ANOVA at P = 0.01 and the hierarchical clustering using the TIGR MeV (MultiExperiment Viewer) v4.1 software, http://www.tm4.org/mev.html. Two replicates were performed. Color coding: red, up-regulated; black, mean; green, down-regulated.
(TIF)

Figure S2 **Alignment of identified pxy-mir-1c with other insect mir-1 registered in the miRBase.** The conserved mature miRNA sequences are highlighted in black.
(TIF)

Figure S3 **Alignment of identified pxy-mir-307 with other insect mir-307 registered in the miRBase.** The conserved mature miRNA sequences are highlighted in black.
(TIF)

Table S1 **Predicted** ***P. xylostella*** **miRNAs using** ***Bombyx mori*** **genome data as reference.**
(XLS)

Table S2 **Predicted** ***P. xylostella*** **miRNAs using its own genome data as reference.**
(XLS)

Table S3 **Primers used for qRT-PCR analysis of 11 miRNAs predicted based on** ***P. xylostella*** **genome data.**
(DOC)
