# Supplementary figures and images for: Identification and Developmental Profiling of microRNAs in Diamondback Moth, Plutellaxylostella (L.)
Source: PLoS One. 2013 Nov 13;8(11):e78787. doi: 10.1371/journal.pone.0078787 (PMC3827265; doi:10.1371/journal.pone.0078787)

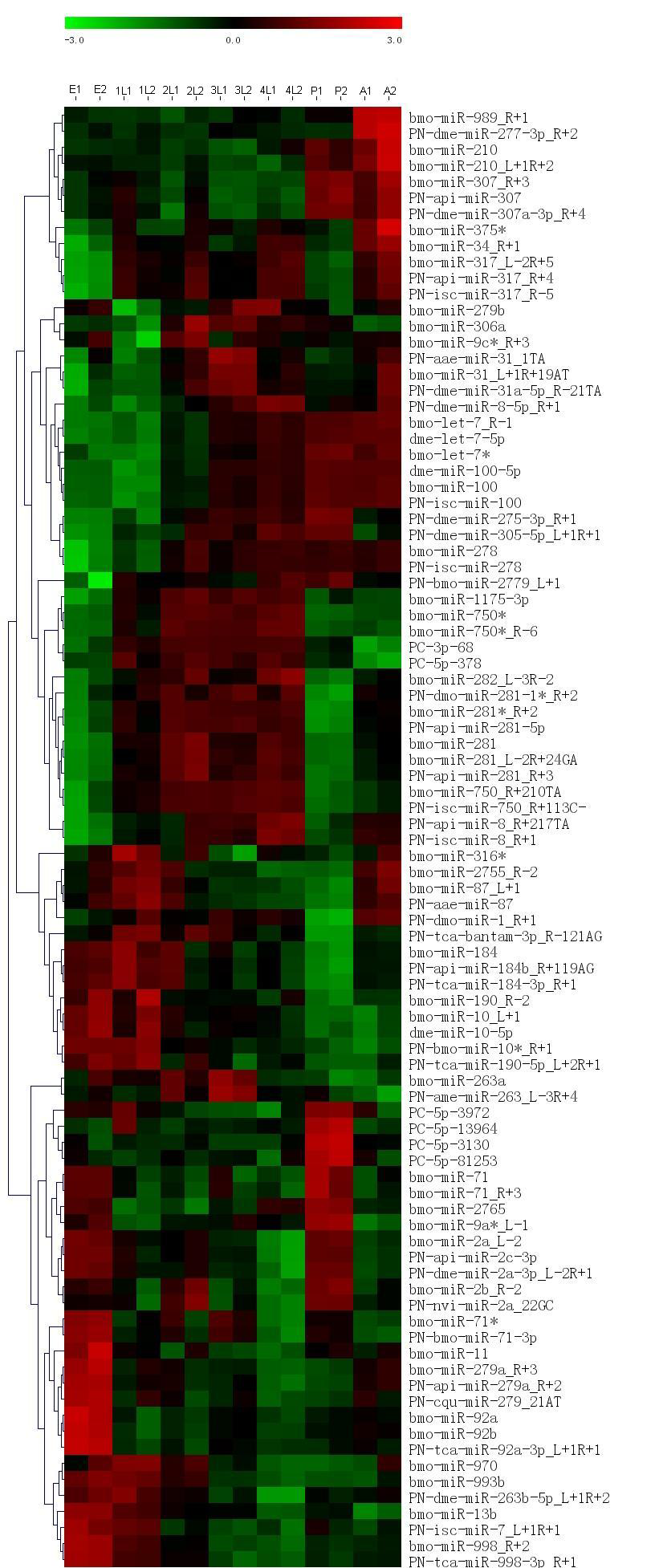

Supplement: Figure S1 — Microarray analysis of expression profiles of predicted P. xylostella miRNAs at different developmental stages. After microarray hybridization and statistical analyses, all detectable P. xylostella miRNAs differentially expressed in eggs, larvae, pupae and adults were subjected to one-way ANOVA at P = 0.01 and the hierarchical clustering using the TIGR MeV (MultiExperiment Viewer) v4.1 software, http://www.tm4.org/mev.html. Two replicates were performed. Color coding: red, up-regulated; black, mean; green, down-regulated. (TIF) [file pone.0078787.s001.tif]

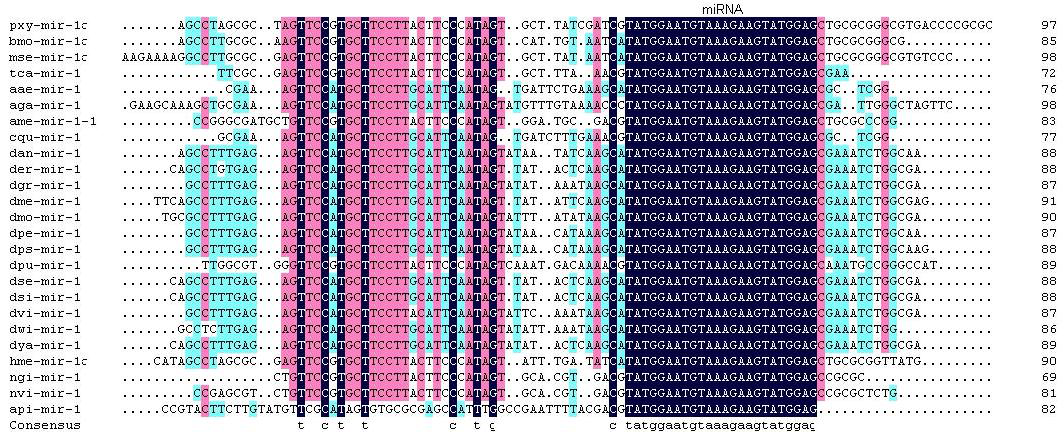

Supplement: Figure S2 — Alignment of identified pxy-mir-1c with other insect mir-1 registered in the miRBase. The conserved mature miRNA sequences are highlighted in black. (TIF) [file pone.0078787.s002.tif]

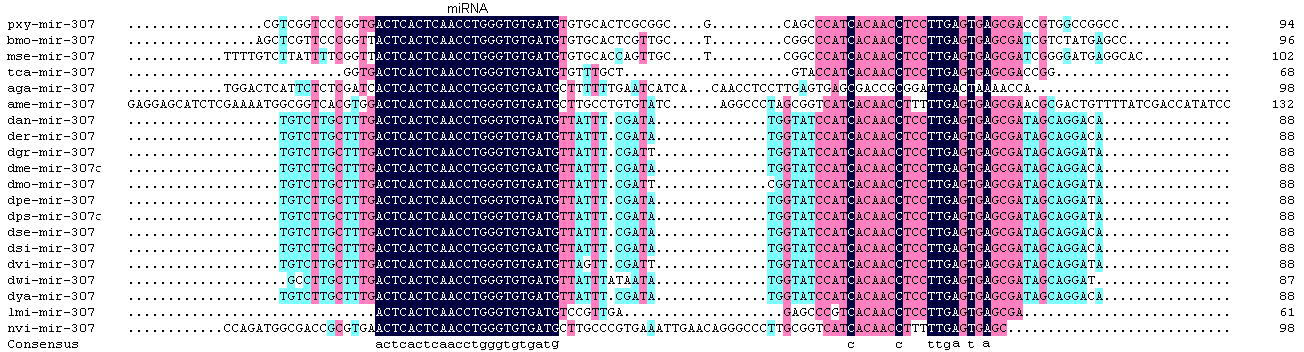

Supplement: Figure S3 — Alignment of identified pxy-mir-307 with other insect mir-307 registered in the miRBase. The conserved mature miRNA sequences are highlighted in black. (TIF) [file pone.0078787.s003.tif]
