# Supplementary material for: Identification and Developmental Profiling of microRNAs in Diamondback Moth, Plutellaxylostella (L.)
Source: PLoS One. 2013 Nov 13;8(11):e78787. doi: 10.1371/journal.pone.0078787 (PMC3827265; doi:10.1371/journal.pone.0078787)
Supplement: Table S3 — Primers used for qRT-PCR analysis of 11 miRNAs predicted based on P. xylostella genome data. (DOC) [file pone.0078787.s006.doc]

Table S3 Primers used for qRT-PCR analysis of 11 miRNAs predicted based on *P. xylostella* genome data

| No. | Gene name | Forward Primer Sequence (5′-3′) | Tm |
| --- | --- | --- | --- |
| 1 | PC-5p-52_2942 | GGCAGTAATTTCCAGATAAACG | 57.8 |
| 2 | bmo-miR-2755-3p | GCACCCTGTCAGACCATACTTG | 58.3 |
| 3 | dme-miR-2a-3p_3ss18AT22GC23CT | ATCACAGCCAGCTTTGTTGAC | 56.9 |
| 4 | dpu-bantam_R-1 | CCGCTGTGAGATCATTGTGA | 57.3 |
| 5 | PC-3p-174_795 | AGTCGCAGGTGAGATGATAGC | 56.8 |
| 6 | bmo-miR-306a-5p_1ss1TC | ACGACCAGGTACTAGGTGACTC | 58.5 |
| 7 | bmo-miR-281-3p_L-2R+2 | CGATGTCATGGAGTTGCTCTC | 55.6 |
| 8 | PC-3p-63_2387 | GCAAATTCAGAGGTAACGGC | 57.4 |
| 9 | PC-3p-61_2483 | CGCCTATCTCAGCTATGTCAC | 58.0 |
| 10 | bmo-mir-6497-p5_1ss10CG | GGGTTTGGAGGGGAAGC | 57.9 |
| 11 | PC-5p-82_1775 | GGCAGAGTCTTGACATGTTCC | 56.2 |
